# Effects of workload, work complexity, and repeated alerts on alert fatigue in a clinical decision support system

**DOI:** 10.1186/s12911-017-0430-8

**Published:** 2017-04-10

**Authors:** Jessica S. Ancker, Alison Edwards, Sarah Nosal, Diane Hauser, Elizabeth Mauer, Rainu Kaushal, with the HITEC Investigators

**Affiliations:** 1 Department of Healthcare Policy & Research, Division of Health Informatics, Weill Cornell Medical College, New York, NY USA; 2 Health Information Technology Evaluation Collaborative (HITEC), 425 E. 61st Street, Suite 301, New York, NY 10065 USA; 3 Department of Family Medicine, Mount Sinai Icahn School of Medicine, New York, NY USA; 4 Institute for Family Health, New York, NY USA; 5 Tehran Heart Center, Tehran University of Medical Sciences, New York, NY USA

**Keywords:** Electronic health records, Clinical decision support, Alert fatigue

## Abstract

**Background:**

Although alert fatigue is blamed for high override rates in contemporary clinical decision support systems, the concept of alert fatigue is poorly defined. We tested hypotheses arising from two possible alert fatigue mechanisms: (A) *cognitive overload* associated with amount of work, complexity of work, and effort distinguishing informative from uninformative alerts, and (B) *desensitization* from repeated exposure to the same alert over time.

**Methods:**

Retrospective cohort study using electronic health record data (both drug alerts and clinical practice reminders) from January 2010 through June 2013 from 112 ambulatory primary care clinicians. The cognitive overload hypotheses were that alert acceptance would be lower with higher workload (number of encounters, number of patients), higher work complexity (patient comorbidity, alerts per encounter), and more alerts low in informational value (repeated alerts for the same patient in the same year). The desensitization hypothesis was that, for newly deployed alerts, acceptance rates would decline after an initial peak.

**Results:**

On average, one-quarter of drug alerts received by a primary care clinician, and one-third of clinical reminders, were repeats for the same patient within the same year. Alert acceptance was associated with work complexity and repeated alerts, but not with the amount of work. Likelihood of reminder acceptance dropped by 30% for each additional reminder received per encounter, and by 10% for each five percentage point increase in proportion of repeated reminders. The newly deployed reminders did not show a pattern of declining response rates over time, which would have been consistent with desensitization. Interestingly, nurse practitioners were 4 times as likely to accept drug alerts as physicians.

**Conclusions:**

Clinicians became less likely to accept alerts as they received more of them, particularly more repeated alerts. There was no evidence of an effect of workload per se, or of desensitization over time for a newly deployed alert. Reducing within-patient repeats may be a promising target for reducing alert overrides and alert fatigue.

## Background

Clinical decision support systems (CDSS), which provide alerts at the point of ordering, can reduce medication errors and adverse drug events [1, 2]. Integrating CDSS into electronic health records (EHRs) allows medication information to be combined with patient information to create alerts about drug-drug interactions, drug allergy contraindications, and other important situations [3]. As a result of this promising history, as well as the federal EHR incentive program (the “meaningful use” program) [4], contemporary EHR products routinely integrate e-prescribing with CDSS. Clinical reminders, such as best-practice alerts to provide preventive services, are also becoming common in EHRs as a result of demonstrated efficacy in improving rates of evidence-based care [5, 6].

In practice, however, 49–96% of alerts are overridden [7], raising questions about the effectiveness of decision support. Although overrides are frequently justified, they can be associated with medication errors and serious adverse events (including death) if clinically important information is inadvertently ignored [7–9]. It is widely accepted that “alert fatigue” explains high override rates [7, 9–14]. However, alert fatigue has been conceptualized in different ways, with different implications.

One conceptual model, which we label ***cognitive overload***, is that alert fatigue is caused by receipt of a large quantity of information along with insufficient time or cognitive resources to distinguish relevant from irrelevant information [7, 15]. Alerts that are not informative contribute to this overload. Uninformative alerts are similar to false alarms, and it is well-established in the human factors literature that false alarms reduce responsiveness to alarms and may also reduce overall performance on tasks interrupted by alarms. [16–18] A second conceptual model of alert fatigue is that repeated exposure to alerts leads to declining responsiveness [9, 19, 20], a phenomenon that can be called ***desensitization*** *.* According to this model, an alert is most effective when it is first noticed, and steadily becomes less effective as the individual becomes acclimatized to it over time.

These definitions are not mutually exclusive, yet they are sufficiently different to suggest different strategies to reduce alert fatigue. If overrides are explained primarily by cognitive overload, response rates could be increased by reducing frequency of uninformative alerts and reducing workload or work complexity. By contrast, if desensitization is a strong factor, response rates could be improved by discontinuing older alerts or changing the presentation of an alert to increase its apparent novelty.

Surprisingly, neither of these hypotheses has been extensively tested, and few effect sizes have been published. Examples of quantitative research supporting the overload explanation include studies finding that override rates decreased overall after irrelevant alerts were discontinued [21], that ability to remember alerts declined as number of different alerts rose [22], and that inappropriate overrides were associated with number of noncritical alerts delivered at the same time [23]. Yet other studies appear to show no evidence of overload. An influential systematic review of CDSS override rates noted that across studies there was no evidence of a relationship between alert volume and percentage overridden [7]. Bryant and colleagues also recently found that alert volume was not correlated with override rate [24]. One study supporting the desensitization explanation showed that response to clinical trial recruiting alerts in the EHR declined by approximately 2.7% per week, falling from about 50% to about 35% over 36 weeks [19]. Many other studies lack physician-level or pre-post data needed to distinguish between the explanations or estimate effect sizes [25, 26], or else report override rates and their predictors without directly addressing alert fatigue [8, 27, 28]. Most work has focused on medication alerts rather than clinical reminders, and has been conducted in academic medical centers.

We had two objectives: (1) to test contrasting hypotheses about alert fatigue arising from the cognitive overload and desensitization models, and; (2) to estimate effect sizes associated with alert fatigue. We conducted the study in an EHR data set from community primary care clinicians that included both medication alerts and clinical reminders.

## Methods

### Design and setting

This retrospective cohort study employed EHR data from the Institute for Family Health (IFH), a network of federally qualified health centers providing safety net care in and around New York City. IFH employs more than 100 physicians and has a patient population of more than 120,000. IFH has been using the EpicCare electronic health record since 2003. Additional details from this study are published elsewhere [29, 30]. The study was approved by the IRBs of Weill Cornell Medical College and IFH.

### Study sample

The study sample included all IFH clinicians who (1) met the criteria for “eligible provider” under the Medicaid meaningful use program, and (2) had at least one patient encounter at IFH between January 1, 2010 and June 30, 2013. We excluded the few IFH clinicians who were not in family practice to increase homogeneity. Data used included encounters with billing diagnoses, alerts fired, and response to each alert. The Johns Hopkins Aggregated Diagnosis Groups (ADG) count of comorbidities was computed on billing diagnoses [31].

In this EHR, best-practice advisories (BPAs) were clinical reminders about recommended care usually presented to a clinician when he or she opened the patient record. These included reminders for preventive services (such as vaccines and cancer screening), disease management (such as lipids testing in diabetes), and compliance with quality initiatives (e.g., reminder to subclassify asthma type to comply with meaningful use quality metrics). BPAs were triggered by patient characteristics (age, sex, diagnosis) at appropriate time intervals (e.g., annually for a flu vaccine reminder, or quarterly for certain diabetes tests). A BPA was considered accepted if the clinician clicked “accept” or opened the order set highlighted in the alert.

The system also included e-prescribing medication alerts. For these e-prescribing alerts, we included only drug-drug interaction (DDI) and drug-allergy interaction (DAI) alerts because other types had very high override rates [29].

### Cognitive overload hypotheses

The cognitive overload hypothesis was that likelihood of accepting alerts would decrease with increases in amount of work, complexity of work, and number of uninformative alerts. No validated measures exist to capture these constructs in EHR data. We therefore developed novel clinician-level markers which were approved by our clinician co-authors as having face validity.

- For amount of work, we included *number of unique patients seen* and *number of encounters per year*. Both of these were intended to provide rough estimate of how much work the clinician did at this organization, and allowed us to compare at an ordinal level clinicians who worked more than other clinicians at this organization.
- As markers of complexity of work, we included *alerts received per encounter* and *comorbidity index* of the clinician’s patients. The comorbidity index was calculated via the Johns Hopkins Aggregated Diagnosis Groups (ADG) algorithm, a well validated case mix metric for ambulatory populations [31]. These 2 indicators were considered likely to be correlated with how complex each encounter was for a clinician, because the existence of multiple comorbidities would require additional evaluation and management, and because it is likely that the additional comorbidities (and corresponding medications) would trigger more alerts for an individual patient.
- As a proxy for low-information alerts, we assessed the proportion of repeated alerts. It would have been ideal to manually review alerts to distinguish appropriate/informative from inappropriate/uninformative alerts, but this was not feasible with the current study, which included more than 1 million alerts. Instead, we captured the *proportion of repeated alerts,* defined as alerts presented to the same clinician for the same patient in the same year. This was because a substantial body of work [7, 8, 32, 33] suggests that repeated CDSS alerts are less likely to be informative (that is, they are often equivalent to false alarms). The human factors literature in other settings shows that high false alarm rates reduce responsiveness as the individual learns, consciously or unconsciously, that the alarm is unreliable [16–18]. Furthermore, some studies suggest that a high rate of false alarms can also reduce overall performance on tasks, due to increased cognitive burden as the individual’s attention becomes divided between relevant and irrelevant information [17]. As a result, we considered it likely that repeated alerts caused additional cognitive overload because of the need to review and dismiss them, even if the clinician tended to dismiss them without reading them.
- For drug alerts only, an additional 2 variables were markers of the complexity of the work: *order sets used per encounter* and *lab tests ordered per encounter*. The rationale for these was the same as the rationale for the complexity of work markers listed above. However, these 2 ordering metrics were included only for the drug alert analyses, because many BPAs contained recommendations to place an order via an order set (e.g., reminders to test hemoglobin A1c in diabetes).

### Desensitization over time hypothesis

We hypothesized that, if desensitization over time occurred, the likelihood of accepting an alert would be highest during the initial months of its deployment, followed by a decline. To test this hypothesis, we selected newly deployed alerts that had sufficient longitudinal information. We restricted the sample to BPAs that were newly launched within the study timeframe, were available continuously for 6 months or more, and were presented to at least 10 clinicians 5 or more times each. This produced a sample of 6 new BPAs and monthly acceptance rates for 46 clinicians which could be graphed and analyzed over time.

### Construction of variables

Analyses were conducted at the clinician level because all hypotheses were at the clinician level. Average alert acceptance rates (outcome variable) were computed per clinician. Patients were attributed to the provider that they saw most frequently. Patient comorbidity scores were computed using the patient’s documented comorbidities the end of each calendar year, and then the average comorbidity for the clinician’s patient panel (using the attribution described in the previous sentence) was calculated. The other covariates were also computed at the clinician level: total number of encounters per year, average number of alerts received per encounter, average number of those alerts that were repeats (i.e., same clinician, same patient, same year), and average number of order sets employed during an encounter.

### Statistical analyses

The primary outcome of interest was the clinician-level alert acceptance rates, which were modeled with negative binomial models. For each model, the model employed an offset which was the natural logarithm of the number of alerts fired. (Additional details on methodology are available in previous papers from the same cohort study [29, 30]). Results are reported as the incident rate ratio (IRR). The incident rate ratio is the alert acceptance rate for clinicians in one category divided by the alert acceptance rate for clinicians in a second category, and it is interpreted more or less like an odds ratio. For example, an IRR of 4.0 for nurse practitioners versus doctors would mean that nurse practitioners were 4 times as likely to respond to an alert as doctors were (see Table 2 for examples). Confidence intervals were calculated from standard errors robust to clustering at the clinician level to account for the correlation of alert acceptances across the 4 years for each clinician [34, 35]. We assessed bivariate and multivariable relationships between the outcome of interest and each of the predictors (nurse practitioner versus physician, attested for meaningful use, female versus male, number of patients seen per year, encounters per year, average alerts received per encounter, average comorbidity count of patients in the panel, percent of all alerts that were within-patient repeats, average number of order sets used per encounter, and number of lab tests ordered per encounter; Table 2). In the multivariable models, one variable was dropped to avoid collinearity; details in Table 2 footnote.

**Fig. 1:**
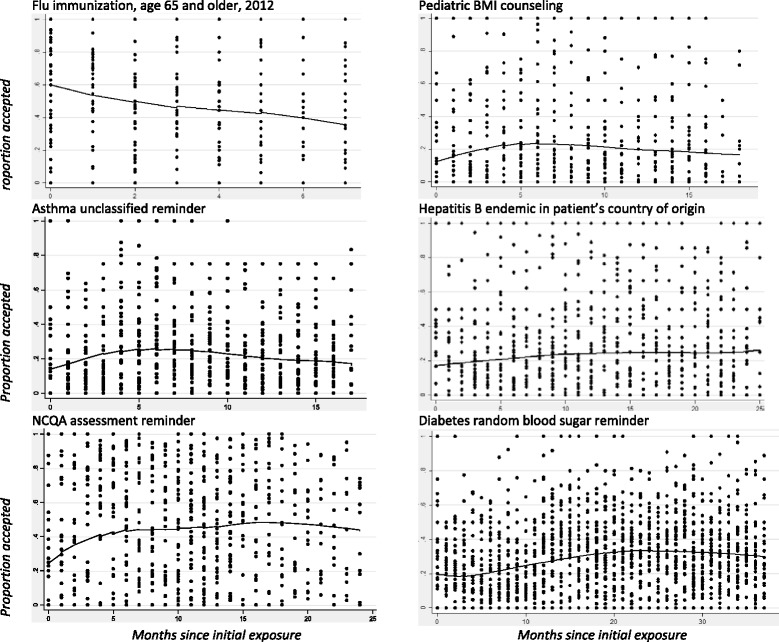
The average acceptance rate across clinicians over time for a specified alert show no clear pattern that would suggest desensitization over time, with the potential exception of the flu immunization alert (see text for interpretation). The curves represent the average acceptance rates (lowess smoothed). Each dot represents one clinician’s acceptance rate for the alert during one month; these are displayed to demonstrate that the alerts included in this analysis had different numbers of clinicians and different numbers of months. The zeros on the x-axis indicate the initial month that the alert was deployed

For the desensitization hypothesis, alert acceptance rates were computed per provider per month, and we then normalized the month such that time zero was the time of alert deployment. Lowess-smoothed graphs are provided showing these rates from time zero through the lifetime of the alert or study end. To model the trend (Fig. 1), slopes over time were estimated with Poisson, zero-inflated Poisson, negative binomial, or zero-inflated negative binomial models (best-fitting model selected on the basis of a likelihood ratio test followed by a Vuong test). For all models, the offset was the log of number of alerts fired. In these models, cluster-robust errors within clinician could not be calculated because some of the clusters (that is, clinicians) had high frequencies of no response (they did not accept any alerts in a given month). Instead, robust standard errors were used [35]. Under the desensitization hypothesis, trendline slopes should have been negative after an initial peak representing high average acceptance rates.

Analyses were performed in SAS 9.3 and Stata 13.

## Results

The sample included 112 clinicians: 93 physicians and 19 nurse practitioners (Table 1). The data set contained 1,266,325 BPAs and 326,203 DDI/DAIs from 430,803 encounters with 99,649 patients. Clinicians typically received more than 4,000 BPAs and 1,000 drug alerts per year (Table 1).

**Table 1:** Characteristics of study sample

	All years	2010	2011	2012	Jan – Jun 2013
Clinicians, n	112	55	70	90	87
Nurse practitioners, n (%)*	19 (17)	6 (11)	8 (11)	12 (13)	15 (17)
Female, n (%)	71 (63)	32 (58)	44 (63)	56 (62)	54 (62)
Attested for meaningful use, n (%)†	42 (38)	33 (60)	42 (60)	42 (47)	41 (47)
Annual patients per clinician, median (Q1-Q3)	791 (406-1269)	968 (424-1336)	896 (429-1341)	758 (354-1337)	521 (391-794)
Annual encounters per clinician, median (Q1-Q3)	1357 (630-2287)	1850 (840-2416)	1695 (834-2562)	1580 (508-2389)	882 (511-1277)
Patient comorbidity score, median (Q1-Q3)‡	0.3 (0.2-0.4)	0.3 (0.2-0.4)	0.3 (0.2-0.4)	0.4 (0.2-0.5)	0.3 (0.2-0.4)
BPAs in use, total	126	93	94	98	93
Annual BPAs received per clinician, median (Q1-Q3)	4227 (2057-6897)	5265 (2739-7652)	5075 (2602-8070)	4310 (1756-7249)	2486 (1625-3799)
Percent BPAs accepted per clinician, median (Q1-Q3)	19.4 (11.8-28.5)	18.6 (13.0-27.0)	19.6 (12.6-30.0)	20.9 (10.7-30.0)	18.6 (10.4-26.0)
DDI/DAI alerts in use, total	3213	2045	1599	1819	1364
Annual alerts received per clinician, median (Q1-Q3)	966 (407-1762)	1116 (580-2169)	1140 (547-2131)	1231 (310-1831)	622 (255-969)
Percent accepted per clinician, median (Q1-Q3)	0.0 (0.0-0.3)	0.0 (0.0-0.4)	0.0 (0.0-0.4)	0.0 (0.0-0.2)	0.0 (0.0-0.2)

*DDI* drug-drug interaction, *DAI* drug-allergy interaction

*All other clinicians are MDs or DOs

†All of these attested during 2012

‡Comorbidity score calculated by Johns Hopkins aggregated diagnosis group algorithm

More than a quarter of BPAs were repeats for the same clinician for the same patient within the same year (median 26.2% per clinician; interquartile range [IQR] 16.5% to 35.1%). Similarly, of all DDI/DAI received by a clinician, almost 1/3 represented repeats for the same patient within the same year (median 31.8% per clinician; IQR 25.6% to 38.2%).

### Cognitive overload hypothesis

For BPAs, acceptance rates went down with increases in the total number of BPAs and repetition of BPAs (Table 2). BPA acceptance rates were also significantly lower with increased average patient complexity (comorbidity count) in the univariate analysis, although this factor narrowly lost significance in the multivariable model (IRR = 0.49, *P* = .06). With every additional BPA per encounter, the likelihood a clinician would accept any BPA dropped by 30% (IRR = 0.70; *p* < .001; Table 2). With each 5% increment in proportion of BPAs that represented within-patient repeats, the likelihood of BPA acceptance dropped by 10% (IRR = 0.90; *p* < .001; Table 2).

**Table 2:** Associations between alert acceptance and clinician characteristics and workload

Predictor	Median value of continuous predictors	Acceptance rates (yes vs. no or above vs. below median)	Bivariate models		Multivariable model	
			IRR (95% CI)	P	IRR (95% CI)	p
For best practice advisories (BPAs)						
Clinician characteristics						
Nurse practitioner (vs physician)	--	22.7% vs 21.0%	1.08 (0.74, 1.57)	.69	0.79 (0.57-1.10)	.16
Attested for meaningful use (vs not attested)	--	22.9% vs 19.3%	1.19 (0.94, 1.51)	.15	1.06 (0.88-1.28)	.50
Female (vs male)	--	23.0% vs 18.4%	1.25 (0.98, 1.59)	.07	1.06 (0.88-1.27)	.54
Markers of workload						
Patients per year	790	20.7% vs 21.7%	1.002 (0.98, 1.02)^c^	.85	---^f^	
Encounters per year	1357	21.8% vs 20.6%	0.999 (0.99, 1.01)^d^	.92	1.01 (1.00-1.02)	.21
Markers of complexity						
BPAs received per encounter	3.2	15.8% vs 26.7%	0.65 (0.55, 0.77)^a^	<.001	0.70 (0.60-0.82)	<.001
Drug alerts received per encounter	0.7	22.7% vs 19.8%	1.17 (0.80, 1.72)^a^	.41	1.91 (1.39-2.64)	<.001
Average ADG comorbidity count of patients	0.3	18.9% vs 23.5%	0.27 (0.10, 0.71)^b^	.008	0.49 (0.23-1.03)	.06
Percent of BPAs that are within-patient repeats	26.2	17.0% vs 25.4%	0.90 (0.87, 0.94)^e^	<.001	0.90 (0.86-0.95)	<.001
For drug alerts (DDI and DAI)						
Clinician characteristics						
Nurse practitioner (vs physician)	--	0.9% vs 0.2%	3.97 (1.64, 9.62)	.002	4.56 (1.72-12.06)	.002
Attested for meaningful use (vs not attested)	--	0.3% vs 0.3%	0.92 (0.40, 2.11)	.85	0.96 (0.49-1.88)	.91
Female (vs male)	--	0.4% vs 0.2%	1.72 (0.82, 3.60)	.15	0.85 (0.37-1.95)	.71
Markers of workload						
Patients per year	790	0.3% vs 0.3%	0.99 (0.94, 1.04)^c^	.60	-----^f^	
Encounters per year	1357	0.3% vs 0.4%	0.98 (0.96, 1.01)^d^	.12	1.00 (0.97-1.04)	.82
Markers of complexity						
BPAs received per encounter	3.2	0.3% vs 0.3%	1.10 (0.78, 1.56)^a^	.59	1.49 (1.03-2.17)	.04
Drug alerts received per encounter	0.7	0.2% vs 0.4%	0.69 (0.29, 1.67)^a^	.42	1.39 (0.44-4.39)	.57
Average ADG comorbidity count of patients	0.3	0.3% vs 0.4%	0.15 (0.03, 0.80)^b^	.03	0.37 (0.05-2.74)	.33
Percent of alerts that are within-patient repeats	31.8	0.3% vs 0.4%	0.87 (0.76, 0.998)^e^	.046	0.84 (0.68-1.03)	.09
Order sets used per encounter	1.1	0.3% vs 0.3%	1.20 (0.44, 3.23)^a^	.72	1.68 (0.76-3.71)	.20
Labs ordered per encounter	0.9	0.3% vs 0.3%	0.89 (0.43, 1.83)^a^	.75	0.80 (0.41-1.56)	.51

^a.^ IRR computed per 1 additional unit

^b.^ IRR computed per 1 additional ADG

^c.^ IRR computed per 100 additional patients

^d.^ IRR computed per 100 additional encounters per year

^e.^ IRR computed per 5 percentage point increment

^f.^ Patients per year omitted from multivariable model to avoid collinearity with encounters per year

NOTE: An IRR (incident rate ratio) is the ratio of 2 rates and is interpreted similarly to a relative risk or odds ratio. For example, the adjusted IRR of 4.56 above indicates that nurse practitioners are more than 4 times as likely to accept drug alerts as physicians are, in a model that controlled for patient comorbidity and proportion of repeated alerts

Associations with similar effect sizes were evident in DDI/DAI but statistical significance was lower because of low acceptance rates (overall less than 1%; Table 2). (Low acceptance rates created what is known as a floor or basement effect, meaning the acceptance rate could not go much lower without hitting 0, analogous to the more familiar ceiling effect, describing situations in which a statistic cannot increase without hitting the upper limit.) Percentage of repeated alerts and patient complexity were both significant predictors of lower alert acceptance rates in the bivariate analysis; in the multivariable model, the effect sizes were similar but the factors lost statistical significance. Also, for DDI/DAI, nurse practitioners had markedly higher acceptance rates than physicians (IRR 4.56, *p* = .002; Table 2).

An unexpected finding was that BPA acceptance was higher among providers who received more drug alerts per encounter (IRR = 1.91; *P* <.001). It is possible that this is due to an unmeasured confounder, for example, polypharmacy at the patient level (which would be likely to be related to complex medical conditions and also lead to higher rates of drug alerts).

Among repeated BPAs (i.e., BPAs presented to the same clinician for the same patient within the same year), if the first instance was overridden, the chance of subsequent instances being overridden was 87.9%. Conversely, if the first instance was accepted, the chance of subsequent instances being overridden was 51.9%. Similarly, among repeated drug alerts, if the clinician overrode the first instance, the chance of overriding subsequent instances was 99.9%, whereas if the first instance was accepted, the chance of overriding subsequent instances was 58.4%.

### Desensitization over time hypothesis

As described in the methods section, there were only 6 newly deployed alerts with sufficient duration and sample size to include in this longitudinal analysis of acceptance rates for individual alerts over time. Only one of 6 alerts showed an early peak in response rate followed by a drop (Fig. 1; slope estimates ranging from +0.16 to -0.34; P values ranging from .03 to .24). This was the 2012 flu immunization reminder for patients over age 65, and it seems likely that the decrease was due to patients reporting that they had received immunizations elsewhere. Two others showed slow rises over the first 6 months followed by slow declines, and the others had no clear temporal pattern.

## Discussion

A clinician’s likelihood of accepting best practice reminders dropped markedly with increases in the number of reminders, number of repeated reminders for the same patient, and overall patient complexity. For drug alerts, acceptance rates showed similar negative correlations with number of repeated alerts and panel complexity, although the low acceptance rates overall led to floor effects and meant that the pattern was weaker. Override rates were not linked to indicators of general workload, such as number of patients seen and number of encounters. For best practice advisories, the clinician’s acceptance rate decreased by about 30% with each additional alert received per patient encounter and by 10% for every five percentage point increment in percent of repeated alerts. Repeats (a specific alert or reminder delivered to one clinician multiple times in a year for the same patient) were extremely common, representing one quarter of the best practice advisories and one third of the drug alerts.

These findings are consistent with the hypothesis that alert fatigue is connected to complexity of work and proportion of repeated (and likely uninformative) alerts. Increased cognitive workload may make it more challenging for clinicians to identify relevant information within a large quantity of less relevant information.

By contrast, the desensitization hypothesis was not supported. When examining the response patterns for newly deployed alerts, we did not observe a decrease in the acceptance rate over time. This argues against the explanation that repeated exposure to the same alert causes acclimatization and alert fatigue. Of 6 new alerts included in this part of the analysis, 1 showed an early peak followed by a negative slope. However, it was an annual flu immunization reminder, and it seems likely that response rate was shaped by seasonality rather than desensitization. Patients presenting late in flu season are more likely to report having received their immunization elsewhere.

The current study contributes to the CDSS literature by testing hypotheses about predictors of alert overrides that arise from different theories of alert fatigue, by examining both medication alerts and clinical reminders, by estimating effect sizes for alert fatigue, and by studying alert fatigue in a meaningful-use era EHR in community settings.

Our findings suggest that responses to textual alerts in EHRs are similar to previously documented responses to audible and visual alarms. [16–18] In other settings, low alarm informativeness and increased cognitive workload predict poor alarm response. Informativeness describes the ability of the alarm/alert to identify an important hazard; an uninformative alert is essentially a false alarm. Response rates may reflect probability matching, a cognitive phenomenon in which likelihood of acting in response to a stimulus becomes calibrated to pay-off likelihood [16, 36]. We could not measure informativeness or false-positive alarm rates directly through manual review in a data set that contain more than 1 million alerts. Instead, we captured the proportion of alerts repeated for the same patient in the same year, finding that one quarter of clinical reminders and one third of drug alerts were repeats. Duplicate alerts have previously been identified as potentially uninformative [7, 8, 32]. Others have previously noted that clinicians are less likely to accept drug interaction alerts for patient who had previously received the same medication [28], which suggests that duplicating the alert at prescription renewal may not provide useful information to the prescriber.

Previous small studies are compatible with the concept of cognitive overload as the combination of low alert informativeness and increased work complexity. For example, a retrospective chart review in which expert reviewers identified inappropriate DDI overrides found the only factor associated with inappropriate overrides to be number of noncritical alerts received at the same time [23]. In a laboratory study, van der Sijs and colleagues invited clinicians to use a simulated system [15]. Under time constraints, 8 of 211 alerts (13%) were handled incorrectly because of skill-based errors (that is, automated actions without conscious attention) that suggested alert fatigue. A pre-post study showed that after irrelevant alerts were retired, pharmacist alert override rates decreased from 93% to 86% [21]. A relevant study outside of health IT was a randomized trial of public health messages delivered by email, fax, or text message. Baseman and colleagues demonstrated that for each 1-message-per-week increase, the odds of recalling message content dropped by 41% [22].

Different hypotheses about alert fatigue suggest different potential solutions. If desensitization were a strong predictor, then response could be improved by discontinuing older alerts or changing presentation to increase salience and apparent novelty. However, we did not find evidence in favor of desensitization. Instead, our study shows strong effects of work complexity and repeated alerts. One approach that is both supported by the current data and potentially feasible is to reduce the frequency of the same alert being delivered to the same clinician for the same patient.

A novel finding of this study is that drug alert acceptance rates were much higher among NPs than among family practice physicians in the same setting. Nurse practitioners have been reported to be more likely to follow guidelines and document care [37, 38]. In addition, NPs may be more likely to accept CDSS recommendations about drugs as a result of having less pharmacology training than physicians [39]. In our study, NPs tended to have less complex patients, but the findings remained statistically significant after controlling for patient complexity, suggesting this difference does not explain the findings.

### Limitations

This was an observational study, and we developed novel metrics because no validated metrics existed for this purpose. The clinicians used a single commercial EHR with interruptive alerts, which might reduce generalizability to other systems or types of alerts. No information was available about severity of DDI or DAI in the alerts, and no manual review was conducted to distinguish appropriate from inappropriate overrides. Many overrides are known to be appropriate, as they are informed by clinical knowledge not captured in structured EHR data [10, 40]. Thus, we cannot conclude definitively that reducing override rates would reduce rate of inappropriate overrides.

We considered alerts that were repeated the same year as potentially uninformative, following the work of previous researchers who have identified repeated alerts as duplicative and likely to have low relevance [7, 8, 32, 33]. However, alert relevance always depends upon the clinical situation.

The best practice advisories studied here generally appeared in the electronic health record upon opening the patient chart, which may or may not have been ideal in terms of clinical workflow and may have contributed to override decisions.

In this secondary analysis of EHR data, we could capture only some of the factors that could influence clinicians. For example, we had no information about ambient noise, interruptions, time pressure, quality of patient-clinician interaction, or multi-tasking. However, this limitation is likely to have biased our findings toward the null. Finally, our participants were exposed to a large number of BPAs and drug alerts, which may have produced ceiling effects for overload as well as desensitization.

As described in the methods, for the desensitization hypotheses, we were forced to use robust rather than cluster-robust errors within clinician. However, even with the robust errors, we rejected the hypothesis, so the additional conservatism of the cluster-robust standard errors (i.e., their greater width) would not have changed our conclusions about the desensitization hypothesis.

## Conclusions

Primary care clinicians became less likely to accept alerts as they received more of them, particularly as they received more repeated (and therefore probably uninformative) alerts. Complexity of the patients was also a factor in bivariate analyses, although not in the multivariable models. These findings are consistent with a model of alert fatigue caused by a high proportion of uninformative alerts combined with complex work that makes it challenging to distinguish relevant from irrelevant alerts. There was no evidence of desensitization or of a general effect of workload. Approaches to reduce the numbers of within-patient repeats could be a promising target for reducing alert override rates and alert fatigue.

## Abbreviations

ADG: Aggregated diagnostic group
BPA: Best-practice advisory
CDSS: Clinical decision support system
DAI: Drug-allergy interaction
DDI: Drug-drug interaction
EHR: Electronic health record
IRR: Incident rate ratio
NP: Nurse-practitioner
