# Correction to: Effects of workload, work complexity, and repeated alerts on alert fatigue in a clinical decision support system

**DOI:** 10.1186/s12911-019-0971-0

**Published:** 2019-11-18

**Authors:** Jessica S. Ancker, Alison Edwards, Sarah Nosal, Diane Hauser, Elizabeth Mauer, Rainu Kaushal, with the HITEC Investigators

**Affiliations:** 1 Department of Healthcare Policy & Research, Division of Health Informatics, Weill Cornell Medical College, New York, NY USA; 2 Health Information Technology Evaluation Collaborative (HITEC), 425 E. 61st Street, Suite 301, New York, NY 10065 USA; 3 Department of Family Medicine, Mount Sinai Icahn School of Medicine, New York, NY USA; 4 Institute for Family Health, New York, NY USA

**Correction to: BMC Medical Informatics and Decision Making (2017) 17:36**

**https://doi.org/10.1186/s12911-017-0430-8**

Following publication of the original article [1], the authors reported that the article erroneously stated that Dr. Ancker was affiliated with the Tehran University of Medical Sciences. Dr. Ancker is not affiliated with that institution.
